# High-Precision Pedestrian Indoor Positioning Method Based on Inertial and Magnetic Field Information

**DOI:** 10.3390/s25092891

**Published:** 2025-05-03

**Authors:** Ning Yu, Xuanhe Chen, Renjian Feng, Yinfeng Wu

**Affiliations:** School of Instrumentation and Optoelectronic Engineering, Beihang University, Beijing 100191, China; nyu@buaa.edu.cn (N.Y.); xuanhchen@buaa.edu.cn (X.C.); rjfeng@buaa.edu.cn (R.F.)

**Keywords:** pedestrian indoor positioning method, magnetic field, fusion localization method, error suppression, trajectory optimization

## Abstract

**Highlights:**

**What are the main findings?**

- Reducing yaw angle errors using magnetic field information and an improved Kalman filter method.
- A trajectory refinement method using magnetic landmarks and pose graph optimization.

**What is the implication of the main finding?**

- The proposed method makes full use of indoor magnetic field information.
- By fusing magnetic heading angle constraints and magnetic landmark matching, the indoor pedestrian localization accuracy is significantly improved.

**Abstract:**

Long-term and high-precision positioning is the key to the pedestrian indoor positioning method. The estimation methods relying only on the inertial measurement unit (IMU) itself lack external observations that can provide absolute information, and the cumulative error easily leads to the distortion of the calculated trajectory. In this paper, based on the Extended Kalman Filter (EKF) algorithm, the environmental magnetic field information is taken as the external observation quantity, and a positioning method combining inertial navigation and the magnetic field is proposed. The cumulative error is suppressed from both the yaw angle and pedestrian pose, and the overall navigation and positioning accuracy is improved. The experimental results show that the proposed fusion method greatly improves the suppression of yaw angle and displacement errors. In a total distance of 297.08 m, the yaw angle error is reduced from 11.043° to 4.778°, and the position error is reduced from 8.999 m to 0.364 m. The relative average error decreases from 3.02% to 0.12%.

## 1. Introduction

With the rapid development of modern times, the demand for accurate navigation and positioning has grown. Standard satellite navigation systems are affected by complex urban environments, and the weak signals received between the satellite and the device often lead to significant positioning errors. Other standard positioning methods, such as those based on Wi-Fi, Bluetooth, ultra-wideband, ultrasound, and radio frequency identification, suffer from high infrastructure requirements, complex initial setup, and high costs. In contrast to these non-autonomous positioning methods, autonomous positioning navigation based on Inertial Measurement Units (IMUs) calculates the trajectory or uses the earth’s magnetic field for positioning, offering advantages such as low cost, high timeliness, and low environmental dependence. However, such problems as accumulated errors during the calculation process and sudden changes in the earth’s magnetic field caused by interference from electronic devices pose challenges to high-precision continuous positioning. The fusion of IMU-based and magnetic field-based positioning methods, which overcomes the drawbacks of inertial navigation and magnetic field navigation in complex urban environments, has become a new development direction for autonomous positioning navigation.

Many researchers have reduced IMU errors and mitigated the interference of the urban environment on magnetic field positioning by optimizing trajectory estimation algorithms, establishing magnetic field fingerprint databases, and conducting fast matching positioning methods, thereby improving the accuracy of autonomous positioning methods. In complex urban districts or indoor environments, satellite positioning systems are limited by the environment, making it difficult to achieve effective data exchange between the device and the satellite, resulting in weak signal reception [1] and signal degradation, which leads to positioning errors. In such environments, common positioning technologies include Wi-Fi [2], Bluetooth [3], Ultra-Wideband (UWB) [4], and Radio Frequency Identification (RFID) [5]. Among them, Wi-Fi and Bluetooth-based positioning technologies are similar [6,7,8,9], both using corresponding fingerprint signals for positioning. The advantage of UWB positioning technology lies in high resolution, low power consumption, etc., and it mainly solves the coordinates of the unknown node based on geometric relationships [10,11]. Additionally, indoor positioning methods are based on infrared technology [12] and ultrasonic technology [13]. The positioning methods mentioned above depend highly on the environment, and some require a prior infrastructure setup. These methods are susceptible to interference in complex environments, leading to positioning errors. Table 1 shows the advantages and limitations of these positioning methods.

Inertial navigation positioning and magnetic field positioning methods are autonomous pedestrian positioning methods that do not require the pre-installation of infrastructure. The inertial positioning method uses only the MEMS-IMU carried by the pedestrian to perform trajectory estimation, thereby obtaining real-time positioning information, with the advantage of being unaffected by environmental interference; the magnetic field positioning method uses the earth’s inherent magnetic field as a reliable information source, with the advantage of having no long-term cumulative errors. For park or indoor scenarios, autonomous positioning methods based on inertial and magnetic field positioning can solve problems like environmental interference, which is common in non-autonomous navigation methods. In applications such as emergency safety and crowd monitoring, these methods do not rely on infrastructure installation, have lower operational costs, and can provide precise personnel positioning in more complex environments, offering broader application prospects and adaptability. However, both autonomous pedestrian positioning methods still face some issues. The inertial navigation positioning method is limited by the size and cost of consumer-grade MEMS-IMUs, leading to more significant random errors; additionally, due to the positioning principle based on integration, the positioning accuracy significantly decreases due to long-term error accumulation. For magnetic field navigation, the accuracy is affected by errors in consumer-grade magnetometers, making it difficult to determine precise locations from magnetic field data. Therefore, magnetic field navigation is often used with other positioning methods. However, the magnetic field environment in parks or indoor settings is relatively complex, and interference from buildings, electronic devices, etc., can cause abrupt changes, making magnetic field data unreliable. Many researchers have already focused on addressing these issues by optimizing the performance of both inertial and magnetic field navigation positioning methods.

In inertial navigation positioning, the positioning algorithms can be divided into two categories: Strapdown Inertial Navigation Systems (SINS) and Pedestrian Dead Reckoning (PDR). The yaw angle and displacement are the two main factors that determine the overall positioning performance of these two methods. In the study of displacement estimation, the Zero-Velocity Update (ZUPT) algorithm is the primary method to correct displacement errors [14]. Wang et al. proposed an innovative adaptive sliding window method that uses Fourier transformation to separate the gait frequency of pedestrians accurately, and adaptively adjusts the detection threshold of walking speed based on the identified frequencies. Compared to traditional methods, this approach reduces false positives and improves detection accuracy [15]. Zhang et al. introduced a foot kinematic model based on an “L”-shaped rigid body to describe the kinematic process during the zero-velocity period. They proposed a foot movement quasi-zero-velocity interval (QZVI) detection method based on peak detection and gait cycle constraints, significantly reducing positioning errors under various movement modes [16]. Chen et al., based on the characteristics of pedestrian inertial data, defined the One-Gait-Cycle (OGC) as its basic unit and proposed an adaptive zero-velocity detector based on optimal interval estimation. They mapped the acceleration to a search space and used hierarchical iterative searches between the zero-velocity reference and the search space to determine the optimal zero-velocity interval (ZVI). This method is more reliable than traditional methods in different scenarios and movement modes [17]. Li et al. proposed a deep learning approach, TCN-Bi-GRU, to learn and distinguish sensor data characteristics during pedestrian walking, enabling more accurate identification of ZVI and optimizing the overall positioning information [18].

In the field of yaw angle error suppression, Niu et al. assumed that pedestrian trajectories on the same floor are closed, using the closure points to control the drift error caused by Foot-INS and applying a smoothing algorithm to distribute the drift error evenly across the entire trajectory [19]. Gui et al. proposed an improved heuristic heading constraint method, which, based on the primary direction heading constraint, uses an algorithm to determine whether the current motion is straight. If not, the correction to the main direction heading is stopped. Their method achieved better results in paths composed of multiple curves, effectively suppressing heading drift [20]. Tian et al. proposed a yaw angle correction method based on a ZVI endpoint smoothing filter. They calculated the yaw angles at each time point in the ZVI using a magnetic sensor and then applied a smoothing filter to obtain the optimal yaw angle. The yaw angle correction was also performed at the ZVI endpoint [21]. To address the issue of yaw angle error accumulation, Wang et al. used indoor environmental information and a finite state machine-decision tree to identify and monitor device modes. They combined map constraints and behavior-aware particle filters to reduce the accumulation of heading errors and improper particle migration, filtering, and correcting erroneous trajectories caused by wall penetration [22].

Magnetic field positioning is an effective positioning method, which is usually integrated with inertial navigation in pedestrian positioning applications. However, in the park or indoor environments, the magnetic field is easily affected by the environment, leading to inaccurate magnetic field measurements. Researchers typically fuse magnetic field information with navigation constraints, such as magnetic heading constraints and magnetic field matching. Chen et al. proposed a heading and position constraint algorithm based on indoor geomagnetic characteristics, using the Quasi-Static Magnetic Field (QSMF) algorithm to divide static and non-static magnetic fields. In the quasi-static magnetic field, magnetic heading and IMU heading are combined to correct the overall heading and reduce heading drift [23]. Zhu et al. designed a deep learning method based on CNN-SVM to detect interference in order to effectively reduce the negative impact of local magnetic field interference on heading estimation. This method improves heading accuracy and positioning precision based on the detection results [24]. Wang et al. focused on indoor environments with significant magnetic field disturbances, combining learned inertial odometry and magnetometer output values. They applied long-term average magnetic heading to reduce the impact of magnetic field disturbances [25]. Kuang et al. proposed a magnetic field matching method that is insensitive to magnetometer bias based on PDR for smartphone-based indoor positioning. By predicting the position, they improved the distinguishability of the magnetic field matching and constrained the search area. Stable positioning results were obtained across different smartphone platforms [26]. Shi et al. proposed the integrated particle filter (IPF) method, integrating inertial navigation with magnetic field matching, assuming all noises follow a Gaussian distribution. The results obtained from the initial position and the IPF are sufficiently reliable. This method reduces the randomness of the PF algorithm and addresses the instability issues of traditional particle filters, improving the overall positioning accuracy [27].

In addition to the optimization methods for yaw angle, displacement, and magnetic field, there are also some integrated approaches. For instance, Li et al. proposed a quality control mechanism based on the interaction between different technologies in a trajectory estimation/magnetic field matching/Wi-Fi fingerprint integration framework. In the first-level filter, errors or unreliable estimates from each individual technology are filtered; in the second-level filter, a threshold-based method is used to set the reliability weight of Wi-Fi through an EKF innovation sequence investigation; in the third-level filter, Wi-Fi and trajectory estimation results are utilized to constrain the magnetic field matching search space, thereby reducing the adaptation rate and lowering the computational load [28]. Zhu et al. developed a collaborative positioning solution based on UWB and Foot-INS. In the solution, an appropriate bias compensation measure was designed to handle NLOS (non-line-of-sight) signals, improving the positioning accuracy of Foot-INS [29]. Yu et al. proposed a method that integrates a Bluetooth positioning system with a trajectory estimation system. The algorithm uses a multi-level constraint-based quasi-static magnetic field detection algorithm for heading and speed estimation while introducing a Dynamic Time Warping (DTW)-based Bluetooth landmark detection algorithm to provide 3D reference positions. An adaptive unscented Kalman filter is employed to fuse multi-source information to ultimately achieve 3D indoor positioning [30]. Yu et al. utilized a MEMS-IMUs network on the foot, calf, and waist to impose lower limb kinematic constraints, reducing step length estimation errors. At the same time, indoor landmark information was used to perform a weighted average of the yaw angles from the three body parts, significantly improving the overall positioning performance for long distances [31].

The optimization methods discussed above address the characteristics of inertial and magnetic field navigation, with targeted optimizations for each. The algorithms optimized for pedestrian inertial navigation only use information from accelerometers and gyroscopes, relying solely on gravity as an accurate external input, without changing the inherent nature of error accumulation. The magnetic field positioning algorithm for pedestrians only utilizes one-sided information from either quasi-static or non-quasi-static magnetic fields, leading to the underutilization of magnetic field data. The inertial navigation and magnetic field fusion algorithm proposed in this paper uses magnetic fields as reliable reference information while separately utilizing quasi-static magnetic field data for yaw angle constraints, non-quasi-static data for yaw angle variable constraints, and magnetic landmark matching. This approach makes more effective use of magnetic field information, resulting in higher precision and more accurate pedestrian walking trajectories. The main contributions of this paper are divided into the following three parts:

1. A fusion yaw angle constraint method based on magnetic field yaw angle is proposed. This method fully utilizes the quasi-static magnetic field environment, incorporating changes in magnetic field strength into the extended Kalman filter as state variables. The magnitude of the covariance function reflects the intensity of the magnetic field changes, which is used to assess the stability of the environmental magnetic field. In the quasi-static magnetic field environment, magnetic field yaw angles are used to constrain the integrated yaw angle from the inertial sensor, thereby improving the accuracy of yaw angle estimation.
2. A fusion yaw angle variable constraint method is proposed based on the magnetic field yaw angle variable. This method is applicable to all magnetic field environments. By analyzing the displacement changes caused by different turning methods during pedestrian turns, the intensity of magnetic field variation relative to displacement is derived. Non-quasi-static magnetic fields are a mixture of multiple local quasi-static magnetic fields. If the magnetic field changes are not drastic relative to displacement, the variation in the magnetic yaw angle can be used to constrain the integrated yaw angle change from the inertial sensor, thereby improving the accuracy of yaw angle estimation.
3. A method for pose constraint using magnetic field landmarks is proposed. This method does not require the collection of the entire floor’s magnetic field map. Still, it instead only collects magnetic field information at points where pedestrians are likely to make turns, creating magnetic landmarks. Using pose and loop closure constraints, the position of pedestrians at magnetic landmark points is constrained, eliminating historical errors. Additionally, the entire pedestrian trajectory can be optimized to match the real trajectory more closely, resulting in high-precision pedestrian positioning.

**Figure 1:**
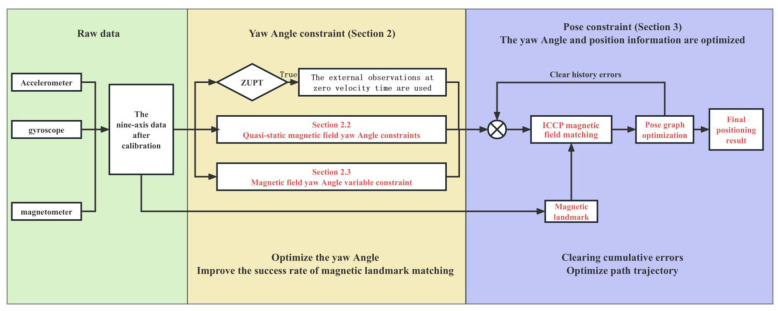
Flowchart of the overall algorithm.

The flowchart of the method proposed in this paper is shown in Figure 1, and the content of the remaining sections is arranged as follows: Section 2 presents the constraint methods for the yaw angle, including Contribution 1 and Contribution 2; Section 3 introduces the optimization method for displacement, i.e., Contribution 3; Section 4 discusses the evaluation results of the proposed fusion method, validated through experiments; Section 5 provides a summary of the work in this paper.

## 2. Magnetic Field-Assisted Yaw Angle Constraint Method

This section proposes a method for constraining the pedestrian yaw angle using magnetic field yaw angle information. First, the SINS algorithm based on a single inertial sensor is introduced, and its results are compared with the method proposed in this section. Section 2.2 and Section 2.3 introduce the first two contributions of this paper.

### 2.1. Pedestrian Positioning Method Based on a Single Inertial Sensor (SINS)

SINS uses the data from the gyroscope to calculate the carrier’s orientation at each sampling time and constructs the Direction Cosine Matrix (DCM). The DCM is then used to map the acceleration data from the body frame (*b*-frame) at each sampling time to the navigation frame (*n*-frame). The acceleration is integrated once to obtain the *n*-system velocity information, and the position information in the *n*-system is obtained by the second integration, as shown in Equation (1).

(1) $$\left\{\begin{array}{c} a^{n}\left(t\right)=C_{b}^{n}\left(t\right)f_{ib}^{b}\left(t\right)-g^{n}\\ v^{n}\left(t+t_{n}\right)=v^{n}\left(t\right)+\int_{t}^{t+t_{n}}a^{n}(\tau )d\tau \\ P^{n}\left(t+t_{n}\right)=P^{n}\left(t\right)+\int_{t}^{t+t_{n}}v^{n}(\tau )d\tau \end{array}\right.$$

In Equation (1), $t_{n}$ represents a time window, $a^{n}$ is the pedestrian acceleration in the *n*-frame, $v^{n}$ is the pedestrian velocity in the *n*-frame, $P^{n}$ is the pedestrian position in the *n*-frame, and $f_{ib}^{b}$ is the output of the accelerometer in the *b*-frame. It is important to note that after mapping the acceleration from the *b*-frame to the *n*-frame, the harmful gravitational acceleration $g^{n}={\left[\begin{array}{ccc} 0 & 0 & g \end{array}\right]}^{T}$ must be subtracted. $C_{b}^{n}$ is the DCM from the *b*-frame to the *n*-frame, and the conversion relationship between quaternion $q={\left[q_{0}\;q_{1}\;q_{2}\;q_{3}\right]}^{T}$ and the DCM is shown in Equation (2).

(2) $$C_{b}^{n}=\left[\begin{array}{ccc} q_{0}^{2}+q_{1}^{2}-q_{2}^{2}-q_{3}^{2} & 2(q_{1}q_{2}+q_{0}q_{3}) & 2(q_{1}q_{3}-q_{0}q_{2})\\ 2(q_{1}q_{2}-q_{0}q_{3}) & q_{0}^{2}-q_{1}^{2}+q_{2}^{2}-q_{3}^{2} & 2(q_{2}q_{3}+q_{0}q_{1})\\ 2(q_{1}q_{3}+q_{0}q_{2}) & 2(q_{2}q_{3}-q_{0}q_{1}) & q_{0}^{2}-q_{1}^{2}-q_{2}^{2}+q_{3}^{2} \end{array}\right]=\left[\begin{array}{ccc} T_{11} & T_{12} & T_{13}\\ T_{21} & T_{22} & T_{23}\\ T_{31} & T_{32} & T_{33} \end{array}\right]$$

**Figure 2:**
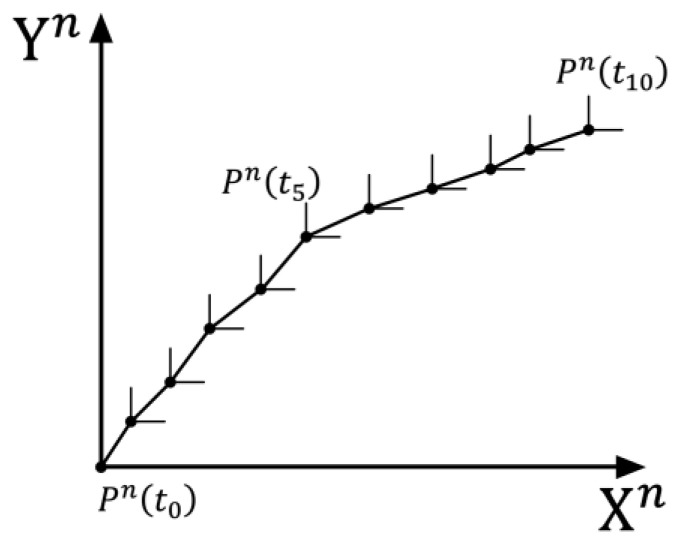
Pedestrian SINS diagram.

The SINS method directly utilizes the output data from the IMU. Its principle is shown in Figure 2, where each point represents a time-discrete sampling point.

Equation (3) provides the method for solving the attitude angle at each sampling time:

(3) $$\left\{\begin{array}{c} \theta ={tan}^{-1}(-\frac{T_{23}}{T_{33}})\\ \gamma ={sin}^{-1}\left(-T_{13}\right)\;\;\\ \psi ={tan}^{-1}(-\frac{T_{12}}{T_{11}}) \end{array}\right.$$

In Equation (3), θ represents the pitch angle, γ represents the roll angle, and ψ represents the yaw angle. By using Equations (1)–(3), the position and attitude angles of the pedestrian at each sampling time can be solved.

**Figure 3:**
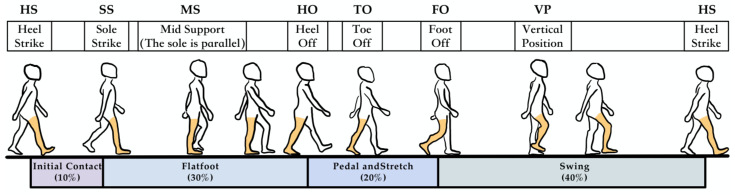
Pedestrian gait cycle diagram.

Pedestrians exhibit a certain periodicity during walking or running, which is more pronounced in the raw IMU data from the lower limb-fixed sensors. Analyzing each gait cycle helps in the subsequent research on inertial navigation and magnetic field fusion positioning. This section decomposes the gait division using the raw IMU data collected, with the cycle and key event markers shown in Figure 3 and Table 2.

In the division of pedestrian gait cycles, the key stages include the following parts:

- **Support Reaction Phase**: This phase spans from the Sole Strike (SS) until the Mid Support (MS). During this phase, the body’s center of gravity continues to rise until it reaches its highest point.
- **Mid Support Phase**: This phase spans from the Mid Support (MS) until the Heel Off (HO). During this phase, the body’s center of gravity starts to descend from the highest point at MS.
- **Post-Support Phase**: This phase spans from the Heel Off (HO) until the Toe Off (TO). During this phase, the body’s center of gravity continues to descend until it reaches its lowest point, while the other foot enters the Heel Strike (HS) phase.

The above gait cycles together form the pedestrian’s zero-velocity interval, and a clear segmentation of the gait cycle can also be observed from the raw data of the inertial sensors.

**Figure 4:**
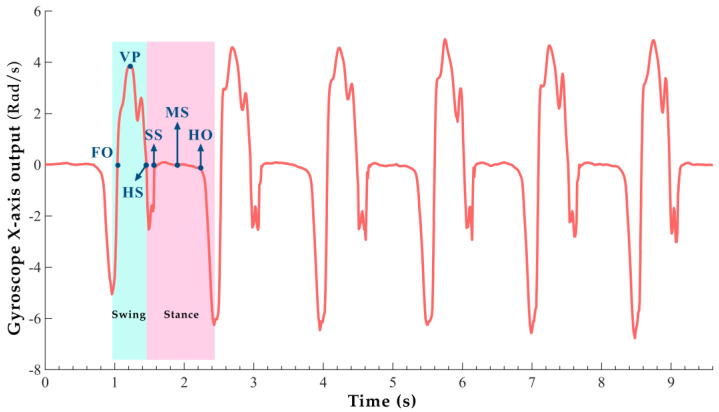
Raw gyro X−axis data gait information.

Figure 4 shows the gait cycle segmentation reflected by the raw gyro *X*-axis data from the foot-mounted IMU. The human body can be divided into two parts from the knee: above the knee, it can only sense the change in the center of gravity, while below the knee, the changes between the support phase and swing phase can be clearly sensed. Additionally, because the gyroscope is sensitive to high-frequency variations, the angular velocity data output from the gyro *X*-axis, fixed on the foot, clearly shows the periodic changes in the pedestrian’s gait during walking. From the figure, it is evident that from the SS moment to the HO moment, the gyro *X*-axis output is nearly zero, reflecting that the pedestrian is in the zero-velocity interval during this period. The proper segmentation and effective recognition of the gait cycle are key points in pedestrian navigation and positioning, and the effective reflection of gait cycle events in sensor data lays a solid foundation for subsequent research.

Due to the working principle of SINS integral, it will lead to the accumulation of errors. When pedestrians use MEMS-IMU for long-term positioning, the error can reach 100 m or more. Therefore, in this paper, we use ZUPT for basic drift suppression and employ the generalized likelihood method to detect ZVI. The generalized likelihood method considers the noise data from the accelerometer and gyroscope and the mean values of their data over a time window. The statistical curve is obtained using these statistics, and the algorithm determines the threshold of the statistical curve to further determine the zero-velocity interval. The formula is as follows:

(4) $$\left\{\begin{array}{c} T_{i}=\frac{1}{N}\Sigma_{j=i}^{i+N-1}\left(\left.\frac{1}{\sigma_{a}^{2}}\right\|a_{j}-g\left.\frac{\bar{a_{i}}}{\left\|\bar{a_{i}}\right\|}\right\|+\frac{1}{\sigma_{\omega }^{2}}{\left\|\omega_{i}\right\|}^{2}\right)<\rho \\ {\bar{a}}_{i}=\frac{1}{N}\Sigma_{j=i}^{i+N-1}(a_{j}) \end{array}\right.$$

In Equation (4), a and ω represent the acceleration data and angular velocity data collected by the MEMS-IMU, respectively, $\sigma_{a}^{2}$ and $\sigma_{\omega }^{2}$ are their variances, i represents the sampling time, g represents gravitational acceleration, and N denotes the time window (the number of sampling points within the window). When the statistic $T_{i}$ is smaller than the decision threshold ρ, it is determined to be a ZVI, with ρ set according to empirical values.

**Figure 5:**
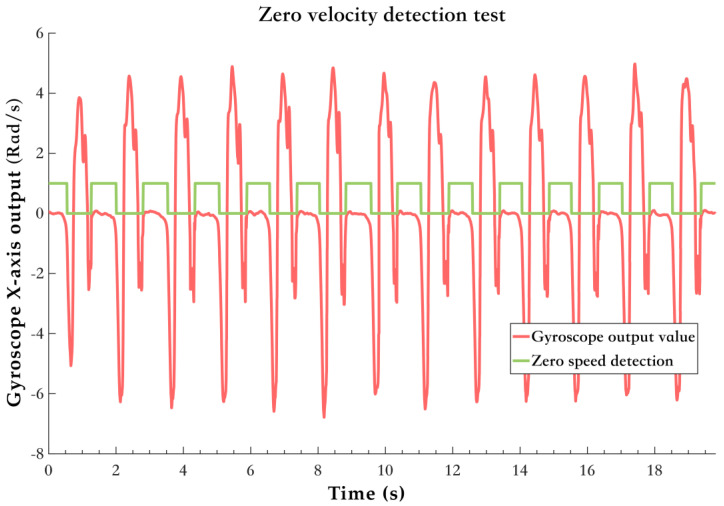
Zero−velocity detection test results.

Figure 5 shows the zero-velocity detection results. The zero-velocity interval corresponds to the flat-foot phase described in Section 2.2. The red curve represents the gyroscope *X*-axis output, as it best reflects the gait information of human movement. The green curve, where the value is 1, indicates the flat-foot phase detected by the algorithm. It accurately reflects the regularity of human movement, corresponding to the SS to HO part in the gait cycle curve.

During the pedestrian positioning process, when in the ZVI, the pedestrian is in a brief “stationary” state, and this period exhibits the following characteristics:

- During the ZVI, the output of the accelerometer in the ***n***-frame is approximately equal to the acceleration due to gravity, with the horizontal acceleration being nearly zero;
- During the ZVI, the position remains unchanged, and the three-dimensional displacement is zero;
- During the ZVI, the pedestrian’s movement velocity is zero.

Based on the above characteristics, the ZUPT method, combined with Kalman filtering, can be employed. This method uses gravity during the ZVI as a measurement to suppress errors, helping mitigate the cumulative drift caused by long-term integration. Furthermore, since only the MEMS-IMU fixed below the knee can accurately detect the flat-foot phase, the subsequent chapters of this paper will focus solely on the case where the sensor is installed on the pedestrian’s foot.

### 2.2. Pedestrian Yaw Angle Constraint Method Based on Magnetic Field Yaw Angle Assistance

In pedestrian positioning scenarios, the displacement of pedestrians is negligible compared to the distance between the earth’s surface and core. Therefore, the change in magnetic field characteristics due to pedestrian displacement can be ignored. Stable magnetic field information can be used to obtain a stable magnetic field yaw angle, which can then be used to constrain the gyroscope yaw angle. However, the environmental magnetic field can disturb the earth’s magnetic field information in environments with interference, leading to more significant magnetic field variations. Thus, this section proposes an improved fusion Extended Kalman Filter (EKF) method, which considers the interference in the magnetic field, making it more versatile.

Identifying quasi-static magnetic fields is a crucial part of the improved fusion algorithm. A quasi-static magnetic field is a magnetic field whose intensity changes little within a certain time and spatial range and can be approximated as a static magnetic field. In practical applications, the three-axis magnetometer in a quasi-static magnetic field can provide a relatively reliable magnetic field yaw angle, which can be fused with the yaw angle provided by the inertial sensors, thereby enhancing the overall reliability of the yaw angle. The environmental magnetic field can be divided into quasi-static and non-quasi-static magnetic fields in pedestrian positioning scenarios. In non-quasi-static magnetic fields, the magnetometer’s magnetic field yaw angle output becomes unreliable due to interference from metals, electronic devices, and other environmental factors.

**Figure 6:**
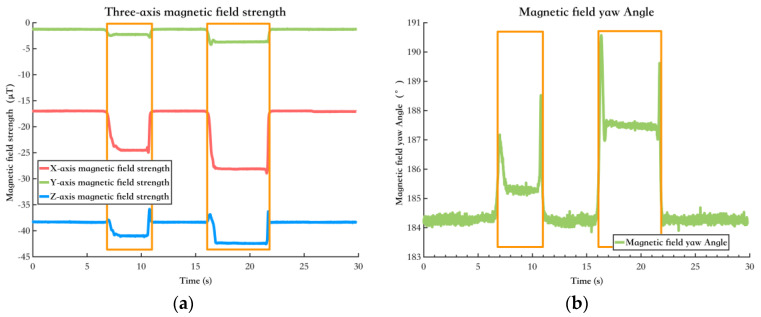
The change in magnetic field information after being disturbed. The data inside the orange box represent the output under interference. (**a**) The change in the output information of the three−axis magnetometer after being disturbed; (**b**) Variation in the magnetic field yaw angle before and after disturbance.

Figure 6 shows the variations in the magnetometer’s output when interfered with in a non-quasi-static magnetic field environment. The data inside the orange box represent the output under interference, while the remaining part shows the output under a quasi-static magnetic field environment. In a non-quasi-static magnetic field, interference significantly affects the magnetic field yaw angle, which can negatively impact the fused yaw angle, decreasing pedestrian positioning accuracy.

The difference between the quasi-static magnetic field and non-quasi-static magnetic fields is the intensity of the changes in the magnetic field sequence. In a quasi-static magnetic field, the changes in the magnetic field sequence are slow over a certain period and space, whereas in a non-quasi-static magnetic field, the changes are more abrupt. Since the pedestrian is continuously moving, the magnetic field data output from the magnetometer can be viewed as a function of time. In the EKF, the rate of change of the magnetic field is included in the state vector. If the intensity of the magnetic field change during a certain sampling period is small, it can be assumed that the magnetic field changes slowly during that period, making it reliable. As a result, the corresponding covariance is small, and the confidence in the magnetic field yaw angle is high. Conversely, if the intensity of the magnetic field change during a certain sampling period is large, it can be assumed that the magnetic field changes significantly during that period, making it unreliable. In this case, the corresponding covariance is large, and the confidence in the magnetic field yaw angle is low.

EKF is widely used in data fusion fields. Due to the generally low sensitivity of MEMS-IMUs in pedestrian positioning, the earth’s rotational angular velocity cannot be used as an observation quantity. Therefore, many studies use gravitational acceleration as the observation quantity, referred to as the “single observation vector” [32]. In this paper, a three-axis magnetometer is incorporated into the fusion scheme, selecting magnetic field information combined with gravity as the “dual observation vector”.

When setting up the filter, the quaternion, the three-axis magnetic field intensity in the n-frame, the rate of change of the three-axis magnetic field intensity in the n-frame, the gyroscope bias, and the accelerometer bias are used to form a 16-dimensional state vector. At the same time, the “dual observation vector” is employed, consisting of gravity and three-axis magnetic field intensity in the n-frame as the observation.

(5) $$\dot{X}=\left[\begin{array}{c} q_{0}-\frac{dtq_{1}\omega_{x}}{2}-\frac{dtq_{2}\omega_{y}}{2}-\frac{dtq_{3}\omega_{z}}{2}\\ q_{1}+\frac{dtq_{0}\omega_{x}}{2}-\frac{dtq_{3}\omega_{y}}{2}+\frac{dtq_{2}\omega_{z}}{2}\\ q_{2}+\frac{dtq_{2}\omega_{x}}{2}+\frac{dtq_{0}\omega_{y}}{2}-\frac{dtq_{1}\omega_{z}}{2}\\ q_{3}-\frac{dtq_{2}\omega_{x}}{2}+\frac{dtq_{1}\omega_{y}}{2}+\frac{dtq_{0}\omega_{z}}{2}\\ m_{x}+dt\Delta m_{x}\\ m_{y}+dt\Delta m_{y}\\ m_{z}+dt\Delta m_{z}\\ \Delta m_{x}\\ \Delta m_{y}\\ \Delta m_{z}\\ 0\\ 0\\ 0\\ 0\\ 0\\ 0 \end{array}\right]$$

Equation (5) is the state-space expression,

(6) $$X={\left[\begin{array}{cccccccccccccccc} q_{0} & q_{1} & q_{2} & q_{3} & m_{x} & m_{y} & m_{z} & \Delta m_{x} & \Delta m_{y} & \Delta m_{z} & \varepsilon_{x} & \varepsilon_{y} & \varepsilon_{z} & \nabla_{x} & \nabla_{y} & \nabla_{z} \end{array}\right]}^{T}$$

Equation (6) represents the 16-dimensional state vector. It should be noted that, compared to traditional pedestrian trajectory estimation algorithms based on Kalman filtering, which only update at zero-velocity moments, the EKF used in this paper also performs observation updates at non-zero velocity moments. Since the pedestrian’s movement speed is relatively slow, although there is motion acceleration interference at non-zero velocity moments, usable information is still available for observation. Therefore, in this method, by calculating the covariance matrix, the covariance matrix of the gravity measurement is scaled up at non-zero velocity moments to reduce its confidence level. This approach maximizes the use of observations while ensuring that the overall error in the pedestrian trajectory estimation system does not increase due to motion acceleration interference.

An experiment was conducted on a square path with a total length of 32.8 m, comparing the fusion EKF method proposed in this section with the conventional EKF method using a single inertial sensor. The comparison results of the yaw angle error are as follows.

### 2.3. Pedestrian Yaw Angle Change Constraint Method Based on Magnetic Field Yaw Angle Variation Assistance

During pedestrian walking, the movement mode can be divided into straight-line walking and turning walking. When pedestrians perform straight-line walking and turning, the primary factor influencing overall yaw angle accuracy is the inaccuracy of the yaw angle estimated by the gyroscope. In the previous section, this paper used the magnetic field yaw angle to constrain the pedestrian’s yaw angle, thereby improving the accuracy of the yaw angle estimate. Meanwhile, if the environmental magnetic field interference is minimal, the magnetic field yaw angle can provide good constraints during both straight-line walking and turning. However, from the perspective of enhancing the applicability of the fusion algorithm, there are better methods for constraining the change in yaw angle during pedestrian turning behavior.

The essence of pedestrian turning can be understood as the change in pedestrian heading occurring simultaneously with the change in displacement, that is, when the pedestrian’s yaw angle changes, how large or small the displacement is.

**Figure 7:**
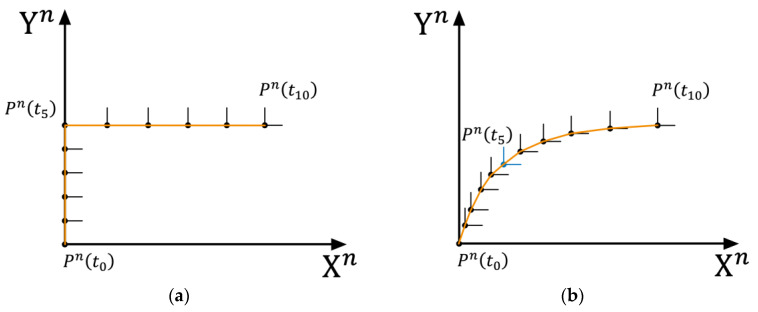
Pedestrian turning action diagram. (**a**) Pedestrian right-angle turn route; (**b**) Pedestrian smooth turn route.

Figure 7 shows a diagram of pedestrian turning behavior, with the orange curve representing the pedestrian’s walking trajectory. It is evident that in both Figure 7a,b, the pedestrian completes the turning behavior between time $t_{0}$ and $t_{10}$. However, in Figure 7a, the turning behavior occurs only at time $t_{5}$ (to emphasize the difference, the turning behavior is extreme in this case). In contrast, in Figure 7b, the pedestrian’s turning behavior is smoothed over the entire time from $t_{0}$ to $t_{10}$. Comparing the turning behavior between Figure 7a and the turning behavior in Figure 7b, both involve a 90° yaw angle change, but the displacement in Figure 7a is zero. On the other hand, the displacement in Figure 7b is the distance between points $P^{n}(t_{0})$ and $P^{n}(t_{10})$. For any point in space $P^{n}$, even if the current environmental magnetic field is interfered with, as long as the interference source is a long-term stable and unchanged source, when the sensor’s orientation changes, if no displacement occurs, the point can be considered as a quasi-static magnetic field point. In an environment where the magnetic field is disturbed, if the pedestrian walks according to the method in Figure 7a, the change in magnetic yaw angle can be used to constrain the change in gyroscope yaw angle, thus achieving higher accuracy and improving overall positioning accuracy.

The filter is set up such that when the fusion algorithm detects a turn, it will evaluate the displacement before and after the turn. If the displacement before and after the turn is large, it is considered that the magnetic field information at this point is unreliable, and the weight is more dependent on the heading angle change calculated from the gyroscope data. Conversely, if the displacement before and after the turn is small, the magnetic field information is considered reliable, and the weight is more inclined toward the heading angle change calculated from the three-axis magnetometer.

(7) $$\frac{dm}{dt}=\frac{dm}{dp}\frac{dp}{dt}=\frac{dm}{dp}v$$

In Equation (7), m represents the three-axis magnetic field strength, and p represents the pedestrian’s position, which indicates that the magnetic field strength is only a function of position. Thus, we have

(8) $$\Delta m=\frac{dm}{dp}v\Delta t$$

The covariance matrix of the state transition noise is used to describe its magnitude. When it is large, the current magnetic field information is considered unreliable, and less trust is placed in the magnetic field information. When it is small, it indicates that the current magnetic field information is reliable, and more reliance is placed on the magnetic field information.

Section 2 proposed an improved EKF method, which corrects inertial sensor data using magnetic field information, further suppressing heading angle errors. The fusion method utilizes the intensity of magnetic field variations to assess the reliability of magnetic field data while also incorporating magnetic yaw angle variable constraints based on the characteristics of pedestrian turning states. The overall yaw angle error is suppressed by fully utilizing the environmental magnetic field information, thus enhancing accuracy. The relevant experimental results will be presented and analyzed collectively in Section 4.2.

## 3. Pedestrian Pose Optimization Method Based on Magnetic Landmark Matching

Section 2 primarily addresses yaw angle errors by proposing an improved fusion EKF algorithm that uses magnetic field information to constrain the yaw angle, aiming to improve overall yaw angle accuracy. However, due to the continuous accumulation of errors in low-cost MEMS-IMUs and the inevitable presence of points with significant changes in indoor or quasi-indoor magnetic fields, the system may still experience error accumulation. Magnetic Field Matching (MM) is one of the mainstream indoor positioning methods that utilizes the natural distribution of magnetic field information.

Indoor or quasi-indoor magnetic fields refer to the combined magnetic field resulting from the earth’s magnetic field and environmental magnetic field interference sources. In Section 2, the environmental magnetic field is divided into quasi-static and non-quasi-static magnetic fields. Section 2 focuses on the full utilization of magnetic heading angle information from quasi-static magnetic fields, while this section focuses on the full utilization of non-quasi-static magnetic field information. Non-quasi-static magnetic fields exhibit significant spatial variability, meaning that the magnetic field changes relatively drastically with position. Although the spatial variability of non-quasi-static magnetic fields prevents the direct use of their magnetic field information (such as magnetic heading angles), these features provide a high-precision absolute positioning method for fingerprint matching. These typical non-quasi-static magnetic fields often appear at corners or doorways in areas with significant amounts of reinforced concrete. In this section, magnetic field information was collected from points with obvious magnetic field features, and a matching algorithm was designed and validated.

### 3.1. Magnetic Landmark Collection and Construction

Landmarks are distinctive features (such as corridor corners, etc.). Due to the characteristics of building structures, map-based landmarks naturally constrain the pedestrian’s trajectory, while MEMS-IMU data at corresponding landmark positions also show specific features. Magnetic landmarks based on magnetic field maps are influenced by building structures, which affect the natural magnetic field, creating unique environmental magnetic fields. These fields are more pronounced near locations such as corridor corners or metal doors, making the magnetic field maps of these locations more distinguishable.

The spatiotemporal stability of the environmental magnetic field is the basis for magnetic landmark matching. The field must remain stable over a certain period to utilize magnetic landmarks in the magnetic field map for matching. Experiments were conducted in indoor open spaces and corridors. Test subjects held magnetic field sensors, walked along the same path, and were tested at different times. The two tests were separated by 24 h.

**Figure 8:**
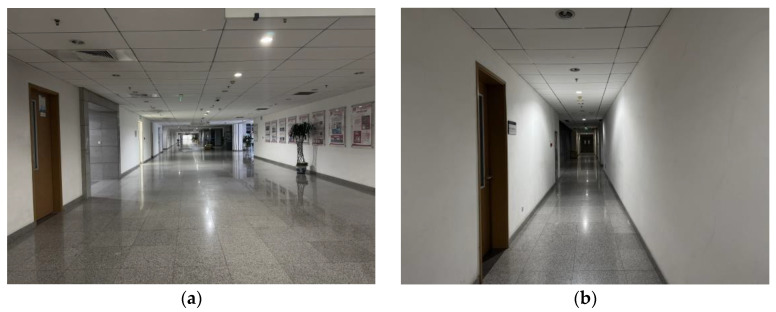
Spatial and temporal stability testing of typical indoor environmental magnetic fields. (**a**) Indoor open space scene; (**b**) Indoor corridor scene.

**Figure 9:**
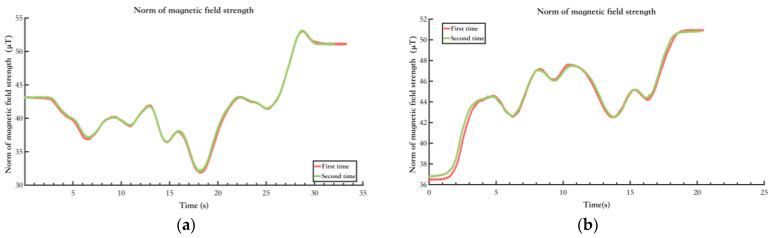
Testing results of spatial and temporal stability of typical indoor environmental magnetic fields. (**a**) Indoor open space scene; (**b**) Indoor corridor scene.

Figure 8 shows the environments of this test, and Figure 9 shows the results. Figure 9a shows the magnetic field features in the environment of Figure 8a, and Figure 9b shows the magnetic field features in the environment of Figure 8b. It can be seen that the magnetic field features in various typical scenarios remain stable and similar across different time periods. Moreover, in the typical experimental scenario of the open indoor space shown in Figure 8b, where pedestrian flow changes over different periods, the impact on the magnetic field features remains minimal. Therefore, it can be concluded that the environmental magnetic field feature distribution in this experiment’s indoor or quasi-indoor scenes exhibits spatiotemporal stability. It should be noted that there were no large-scale changes in the indoor environment in the test setting. Building materials such as steel reinforcement and cement are the primary causes of changes in indoor magnetic fields. If large-scale changes in the indoor environment occur, the indoor magnetic field will inevitably change, and the aforementioned spatiotemporal stability will no longer hold.

Magnetic landmark matching positioning mainly consists of two stages: the offline training stage and the online positioning stage. The offline training stage refers to the process of collecting magnetic field information to establish a one-to-one correspondence between the navigation coordinates and the magnetic field information. The online positioning stage refers to the process of searching for the magnetic landmark fingerprint information in the fingerprint database that has the highest similarity to the measured fingerprint, ultimately determining the position in the navigation coordinate system.

Magnetic landmark fingerprint information collection mainly includes the collection of position data in the navigation coordinate system and the collection of environmental magnetic field information. Position information can be collected by manually calibrating reference points or using MEMS-IMUs; magnetic field information is collected using a three-axis magnetometer. The collection methods can be divided into three categories: point-based collection, continuous collection [33], and pedestrian trajectory estimation collection. This section uses both point-based collection and pedestrian trajectory estimation collection. The point-based method roughly describes the magnetic field profile around the magnetic landmark, while the pedestrian trajectory estimation method, combined with a few calibration points, refines the environmental magnetic field characteristics near the pedestrian path.

In the construction of the environmental magnetic field map, if a local precise magnetic field map describing magnetic field changes is required, spatial interpolation methods must be used. This section uses Kriging interpolation for constructing the magnetic landmark fingerprint database, and the method is as follows:

(9) $$z_{k}=\sum_{i=1}^{n}\omega_{i}z_{i}$$

In Equation (9), $z_{k}$ represents the estimated value of the interpolation point; $z_{i}$ is the attribute value of the *i*th sample point from a total of n known points. The known points refer to the sample points where the magnetic field intensity information is known, and the unknown points refer to the points within the same measurement area for which the magnetic field intensity has not yet been collected. The distance between known points and an unknown point is typically computed using the Euclidean distance. $\omega_{i}$ is the weight coefficient for the known point. In the Kriging interpolation method, the weight values of each sample point to the interpolation point are critical to its accuracy. The weight $\omega_{i}$ should be chosen to ensure that the interpolation is an unbiased estimate, and its variance should be smaller than the variance generated by other linear combinations of sample values. The value of $\omega_{i}$ should satisfy the following equation:

(10) $$\left\{\begin{array}{l} \sum_{i=1}^{n}\omega_{i}=1\\ \sum_{j=1}^{n}\omega_{i}\gamma \left(h_{ij}\right)+\lambda =\gamma \left(h_{il}\right),\;\;i=1,2,\cdots ,n\;\;\;\; \end{array}\right.$$

In Equation (10), $\gamma (h_{ij})$ represents the variation function between known points i and j, $\gamma (h_{il})$ represents the variation function between the known point i and the unknown point l, and λ is the Lagrange coefficient.

The main steps of the Kriging interpolation method are as follows:

(1) Input the measured raw data, which in this paper includes magnetic field and position data;
(2) Grid the interpolation area and divide it into appropriate grid sizes;
(3) Use the theoretical variogram model to fit the experimental variogram values, and simultaneously substitute the theoretical variogram into Equation (10) to solve for the weight coefficient $\omega_{i}$;
(4) The final step is to substitute the weight coefficient obtained from Equation (10) into Equation (9) to solve for the data values at the unknown points.

In this paper, the calculation formula for the variation function of the Kriging interpolation method is as follows:


(11) $$\gamma (h)=\frac{1}{2N}\sum_{i=1}^{N}{\left[z(x_{i})-z(x_{i}+h)\right]}^{2}$$

In Equation (11), $z(x_{i})$ and $z(x_{i}+h)$ represent the magnetic field feature information at points $x_{i}$ and $x_{i}+h$, respectively. h represents the lag distance, and N represents the number of paired measurement points within the lag distance.

The theoretical variation model uses a Gaussian model, as shown in Equation (12). The h is the lag distance, $C_{0}$ is the nugget, C is the scale, and $\sqrt{3}a$ is the range.

(12) $$\gamma (h)=\left\{\begin{array}{ll} 0 & h=0\\ C_{0}+C\left(1-e^{-h^{2}/a^{2}}\right) & h>0 \end{array}\right.$$

### 3.2. Magnetic Field Matching Algorithm

The magnetic field matching algorithm is the core of the magnetic field matching algorithm based on magnetic landmarks. It refers to the process of searching and comparing the magnetic field sequence to be matched within a known magnetic field area. The algorithm’s positioning accuracy and matching efficiency mainly depend on the accuracy and time of the magnetic field sequence search and matching.

The essence of the Iterative Closest Contour Point (ICCP) algorithm is the Iterative Closest Point (ICP) algorithm in image matching. The basic principle of the algorithm is that, under the condition that the relative position of each point in the trajectory output by inertial navigation remains unchanged, the algorithm iterates by translating and rotating the inertial navigation output trajectory so that the transformed trajectory’s corresponding magnetic field data sequence fits as closely as possible to the contour lines of the actual data points measured by the three-axis magnetometer. Thus, the trajectory is determined to be the best matching trajectory. The ICCP algorithm also considers pedestrian (carrier) position errors and heading errors, offering a broad range of applicability.

**Figure 10:**
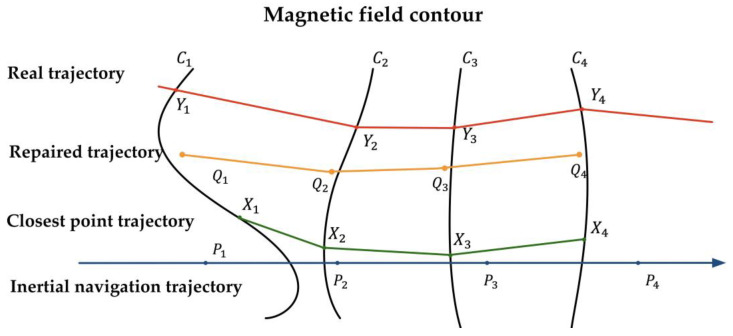
ICCP algorithm schematic diagram.

Figure 10 shows the schematic diagram of the ICCP algorithm, and its specific implementation process is as follows:

(1) When the pedestrian enters the area to be matched, the magnetic field feature data sequence obtained from the actual measurements by the magnetic field sensor is
  (13) $$H_{m}=\left\{m_{Y_{k}},k=1,2,\cdots ,n\right\}$$
  search for the magnetic field contour line $C_{k}$ corresponding to each magnetic field feature data value $m_{Y_{k}}$ on the magnetic field map;
(2) Use the corresponding $P_{k}$ as the center, apply the Euclidean distance as the evaluation criterion, and search for the point on the corresponding contour line $C_{k}$ that is closest to $P_{k}$, denoted as $X_{k}$. At the same time, calculate the rigid transformation matrix between $P_{k}$ and $X_{k}$;
(3) Perform a rigid transformation on the inertial navigation trajectory point $P_{k}$ and correct it to obtain the updated iterative position point$\;Q_{k}$. Then, continue using Euclidean distance as the evaluation criterion to find the point on the repaired trajectory that is closest to the magnetic field contour line $C_{k}$;
(4) Repeat steps (1) to (3) for iterative calculation. When the termination condition for iteration is met, output the best matching trajectory.

The core of the ICCP algorithm process is the rigid transformation matrix $\left[\left.R_{k}\right|T_{k}\right]$, where $R_{k}$ represents the rotation matrix, and $T_{k}$ represents the translation matrix, with the following relationship:

(14) $$X_{k}=\left[R_{k}|T_{k}\right]P_{k}=R_{k}P_{k}+T_{k}$$

This paper uses the quaternion method to solve the rotation matrix $R_{k}$. Meanwhile, in magnetic field matching, the earth’s latitude and longitude only change in the two-dimensional plane, so the rotation matrix $R_{k}$ can be expressed as

(15) $$R_{k}=\left[\begin{array}{cc} cos\theta & -sin\theta \\ sin\theta & cos\theta \end{array}\right]$$

According to Equation (15), to solve for the rotation matrix $R_{k}$, only the rotation angle θ is needed. Therefore, the cross-covariance matrix is constructed for solving it:

(16) $$S=\sum_{k=1}^{n}\omega_{k}\left(Q_{k}-\tilde{Q}\right){\left(P_{k}-\tilde{P}\right)}^{T}$$

In Equation (16), $\tilde{Q}$ and $\tilde{P}$ represent the centroid points of the two sets, and the symmetric matrix W is constructed as

(17) $$W=\left[\begin{array}{cccc} S_{xx}+S_{yy} & 0 & 0 & S_{xy}-S_{yx}\\ 0 & S_{xx}-S_{yy} & S_{12}+S_{21} & 0\\ 0 & S_{xy}+S_{yx} & S_{22}-S_{11} & 0\\ S_{xy}-S_{yx} & 0 & 0 & -S_{xx}-S_{yy} \end{array}\right]$$

In Equation (17), $S_{xy}=\sum_{k=1}^{n}Q_{k}^{\prime }(x_{k}^{\prime })P_{k}^{\prime }(y_{k}^{\prime })$, $S_{yx}$, $S_{xx}$, and $S_{yy}$ have the same principle, as do $Q_{k}^{\prime }\left(x_{k}^{\prime },y_{k}^{\prime }\right)=Q_{k}\left(x_{k},y_{k}\right)-\tilde{Q}$ and $P_{k}^{\prime }\left(x_{k}^{\prime },y_{k}^{\prime }\right)$. The eigenvalues of the matrix are

(18) λ=±Sxx+Syy2+Sxy+Syx212

According to the mathematical principles of ICCP, the eigenvector corresponding to the maximum value of the eigenvalue λ is q, and its eigenvector can be obtained:

(19) $$\left(S_{xx}+S_{yy}-\lambda_{max}\right)q_{0}+\left(S_{yx}-S_{xy}\right)q_{3}=0$$

From Equation (19), θ can be derived, and thus the rotation matrix $R_{k}$ and translation matrix $T_{k}$ can be obtained. Through these calculations, the rigid transformation matrix can be determined. Using the rigid transformation matrix, the new iterative point $Q_{k}$ on the corrected trajectory can be obtained from the position point $P_{k}$ in the inertial navigation trajectory.

### 3.3. Pose Graph Optimization Algorithm

Pose Graph Optimization (PGO) is an important method for back-end nonlinear optimization in Simultaneous Localization and Mapping (SLAM) algorithms. In the field of robot autonomous localization and navigation, it mainly solves the robot’s absolute pose by utilizing relatively noisy observation data provided by the front-end visual odometry. This involves simultaneously solving for the robot’s rotational direction and position at a given sampling point in time [34,35]. In the application environment of this paper, the pose graph optimization method is applied to optimize the pose of the pedestrian inertial navigation and magnetic field fusion algorithm based on the ICCP algorithm. Using the position and heading information from the “repaired trajectory” output by ICCP, loop closure observations are provided as constraints for the PGO method. This allows for optimizing the entire trajectory information, ultimately achieving more accurate pedestrian trajectory estimation performance and suppressing yaw angle and position errors.

The directed graph G=(V,E), composed of various sampled pose points and corresponding measurement edges, is the pose graph. The pose graph consists of “nodes” and “edges”. The “nodes” represent the poses of the pedestrian at the current sampling time, and the “edges” represent the pose constraint relationships between two sampling times. In this paper, $V=\left\{V_{0},V_{1},V_{2},\cdots ,V_{n}\right\}$, where $V_{k}=\left[x_{k},y_{k},\theta_{k}\right]$ denotes the Kth node, k=1,2,⋯,n; here, $\left(x_{k},y_{k}\right)$ represents the position of the pose point in the two-dimensional plane, and $\theta_{k}$ represents the yaw angle of the pose point. $E=\left\{e_{0},e_{1},e_{2},\cdots ,e_{m}\right\}$, where $e_{l}=\left[\theta_{k}^{l},x_{kl},y_{kl}\right]$ represents the lth relative measurement edge, l=1,2,⋯,m, $\theta_{k}^{l}$ represents the relative heading measurement between pose points K and l, and $\left(x_{kl},y_{kl}\right)$ represent the relative position measurement between pose points K and l.

**Figure 11:**
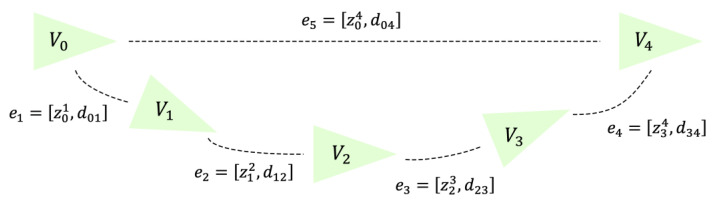
Pedestrian pose graph schematic diagram.

The definitions of the symbols above provide a very intuitive description of the basic concept of the pose graph used in this paper. However, for the sake of computational convenience and to address issues related to notation, we describe the “nodes” and “edges” as $V_{k}=\left[P_{(PGO)k},R_{(PGO)k}\right]$ and $e_{l}=\left[z_{k}^{l},d_{kl}\right]$, respectively. $P_{(PGO)k}$ represents the position of the pose point in the two-dimensional plane, $R_{(PGO)k}$ represents the rotation matrix corresponding to the yaw angle $\theta_{k}$. $z_{k}^{l}$ represents the noisy relative orientation measurement between pose point K and l, which represents the rotation matrix of $\theta_{k}^{l}$, and $d_{kl}$ represents the noisy relative position measurement between pose points K and l. The pedestrian pose graph is shown in Figure 11.

As shown in Figure 11, the pedestrian pose graph schematic diagram is presented. In the diagram, $V_{0}$ represents the pedestrian pose origin, and $V_{4}$ is the “true value” point derived through magnetic landmark magnetic field matching. The edge $e_{5}=\left[z_{0}^{4},d_{04}\right]$ represents the constraint relationship between the two pose points as the “loop closure constraint.” Meanwhile, the edges $e_{1}=\left[z_{0}^{1},d_{01}\right]$, $e_{2}=\left[z_{1}^{2},d_{12}\right]$, $e_{3}=\left[z_{2}^{3},d_{23}\right]$, and $e_{4}=\left[z_{3}^{4},d_{34}\right]$ obtained through pose points $V_{1}$, $V_{2}$, and $V_{3}$ to $V_{4}$ represent the constraint relationships between the pose points as the “recursive constraints.” It is easy to observe that the pose point $V_{4}$ derived from magnetic landmark matching should coincide with the $V_{4}$ obtained from the integrated trajectory estimation algorithm. At this point, the pose of each recursive pose point can be optimized using graph optimization algorithms, thereby optimizing the entire pedestrian walking trajectory and suppressing the cumulative errors in the trajectory.

The pose graph optimization problem refers to estimating the absolute pose from a set of relative measurement values, and the observation equation is shown in Equation (20).

(20) $$\left\{\begin{array}{l} z_{k}^{l}=R_{(PGO)k}^{l}R_{(PGO)noise}=R_{(PGO)l}^{T}R_{(PGO)k}R_{(PGO)noise}\\ d_{kl}=R_{(PGO)l}^{T}\left(P_{(PGO)l}-P_{(PGO)k}\right)+d_{noise}\;\;\;\; \end{array}\right.$$

In Equation (20), $R_{noise}$ represents the heading noise, which follows a von Mises distribution with mean parameter $I_{2\times 2}$ and variance $\omega_{i}$. $d_{noise}$ represents the position noise, which follows a multi-variate Gaussian distribution with mean $O_{2\times 2}$ and variance $\lambda_{i}^{-1}I_{2\times 2}$, and O is the zero matrix.

To solve for the position $P_{k}$ and heading $R_{k}$ of n-th pose points, the algorithm’s goal, according to Equation (20), is to minimize the objective function

(21) $$f=f_{rec}+f_{loop}=\sum_{e_{i}\in E}[\lambda_{i}{∥d_{kl}^{rec}-R_{(PGO)l}^{T}\left(P_{(PGO)l}-P_{(PGO)k}\right)∥}^{2}\;+{\omega_{i}∥z_{k}^{l,rec}-R_{(PGO)l}^{T}R_{(PGO)k}∥}_{F}^{2}]\;\;\;+\sum_{e_{j}\in L}[\gamma_{j}{∥d_{mn}^{loop}-R_{(PGO)m}^{T}\left(P_{(PGO)n}-P_{(PGO)m}\right)∥}^{2}\;+\eta_{j}{∥z_{m}^{n,loop}-R_{(PGO)n}^{T}R_{(PGO)m}∥}_{F}^{2}]$$

In Equation (21), E denotes the set of all recursive constraint edges, and L denotes the set of all loop closure constraint edges. $\lambda_{i}$ represents the weight coefficient for the translation term of the recursive constraints, while $\omega_{i}$ denotes the weight coefficient for the rotation term of the recursive constraints. Similarly, $\gamma_{j}$ represents the weight coefficient for the translation term of the loop closure constraints, and $\eta_{j}$ represents the weight coefficient for its rotation term.

This section uses the Trust-Region Algorithm to solve the problem of minimizing the objective function.

In Section 3, we employ ICCP-based magnetic field matching along with the PGO method for overall trajectory refinement to eliminate cumulative errors. By performing local matching at magnetic landmark locations, a locally repaired trajectory is obtained. The position and yaw information from this locally repaired trajectory serve as loop closure observations for the PGO method, further eliminating historical errors.

## 4. Experiment and Results

This section provides experimental validation of the proposed method in this paper, which integrates magnetic field information to correct the trajectory and suppress errors in yaw angle and displacement. The comparison and analysis of the overall positioning accuracy are also conducted.

### 4.1. Experimental Environment and Setup

**Figure 12:**
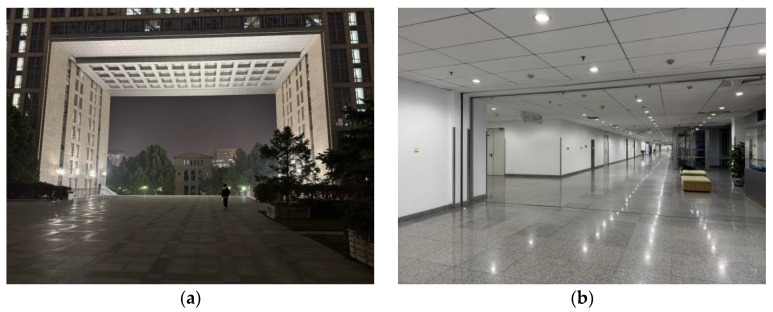
Experimental sites. (**a**) Second-floor platform of the teaching building; (**b**) Indoor area of the teaching building.

Considering the application environment of the fusion algorithm in this paper, indoor and quasi-indoor scenes are selected, as shown in Figure 12.

Figure 12a shows the second-floor platform of the teaching building, representing a quasi-indoor environment as described in this paper. Since other buildings are under it, it is affected by magnetic interference from building materials, making it a quasi-indoor environment with relatively small magnetic interference. Figure 12b shows the indoor environment, which, compared to the quasi-indoor experimental site mentioned earlier, has more, and more complex, sources of magnetic field disturbances.

**Figure 13:**
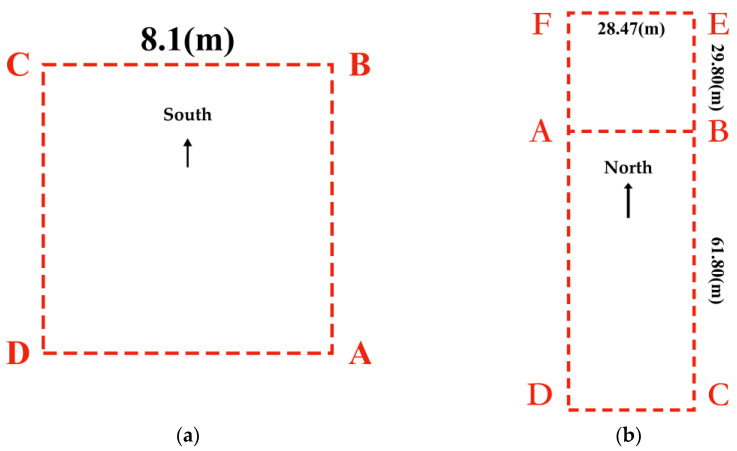
Walking experiment route. (**a**) Experiment route 1; (**b**) Experiment route 2.

Figure 13 shows the walking experiment route. Figure 13a is the experimental route in the quasi-indoor environment of Figure 12a, a square path with each side measuring 8.1 m and a total length of 32.8 m. This route is used to experimentally validate the fusion algorithms based on magnetic yaw angle constraints and yaw angle variable constraints described in Section 2. Figure 13b shows the experimental route in the indoor environment of Figure 12b, which follows an 8-shaped route. The test subject starts from point A and walks in the sequence A-B-C-D-A-B-E-F-A. The length of the AF segment is 29.8 m, the length of the AB segment is 28.47 m, and the length of the AD segment is 61.8 m, making the total walking distance 297.08 m. This experimental route is primarily used to analyze the effect of the magnetic landmark matching fusion algorithm described in Section 3 and to validate and analyze the overall performance of the fusion algorithms proposed in Section 2 and Section 3.

**Figure 14:**
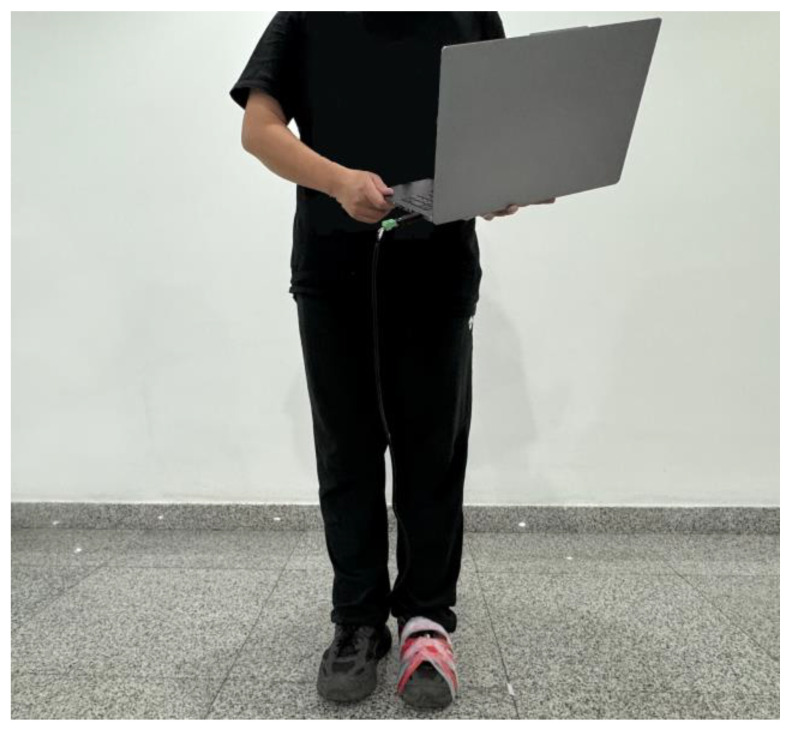
The test subject holds a computer connected to the IMU on the foot.

Based on the analysis of pedestrian gait cycles in this paper, it is shown that for the fusion method proposed in this paper, the sensor should be mounted below the pedestrian’s knee to achieve the best gait validation, thereby providing better localization performance. Therefore, in all experiments in this paper, the sensors are rigidly mounted on the feet of the test subjects, as shown in Figure 14.

The sensor used in this paper is the HWT905-TTL(WitMotion Shenzhen Co., Ltd, ShenZhen, China), which includes a three-axis gyroscope, three-axis accelerometer, and three-axis magnetometer. Table 3 shows some parameters from the official sensor manual.

### 4.2. Optimization Algorithm Experimental Validation

Based on the experimental route diagram presented in Section 4.1, the optimization algorithms proposed in Section 2 and Section 3 were experimentally validated, demonstrating the improvement effect of magnetic field information on pure inertial navigation positioning results.

**Figure 15:**
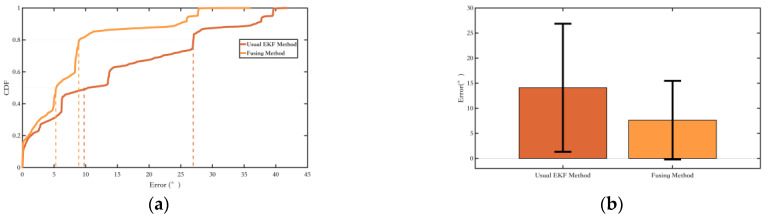
Comparison of the fusing method and the conventional EKF method in Section 2.2. (**a**) The probability of the yaw error distributions of different methods; (**b**) The yaw error bars of different methods. The range indicated by the black line represents the RMS value of the corresponding error.

Firstly, an experiment was conducted on Route 1 with a total length of 32.8 m to validate the method proposed in Section 2.2, which constrains the pedestrian yaw angle based on the magnetic field yaw angle. The comparison results of the yaw angle error are as follows:

From the yaw angle error plot as Figure 15, it can be observed that the fusion EKF method, which integrates inertial navigation and magnetic field, shows a significant improvement in suppressing yaw angle errors compared to the conventional EKF method, with the yaw angle error further reduced.

**Figure 16:**
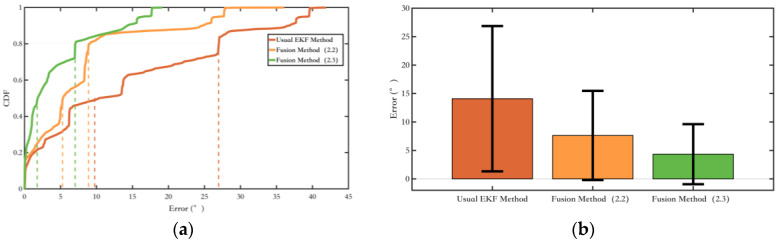
Comparison of the fusing method and the conventional EKF method in Section 2.3. (**a**) The probability of the yaw error distributions of different methods; (**b**) The yaw error bars of different methods. The range indicated by the black line represents the RMS value of the corresponding error.

Secondly, the experiment was conducted on Route 1 to validate the method proposed in Section 2.3, which constrains the change in pedestrian yaw angle using magnetic field yaw angle variation assistance. The yaw angle error results are shown in Figure 16.

As shown in the yaw angle error plot in Figure 16, the yaw angle error suppression method proposed in Section 2.3 has made further improvements compared to the method presented in Section 2.2, with a more significant effect on suppressing yaw angle errors. Additionally, it has expanded its applicability by considering the magnetic heading angle variation during pedestrian turns.

**Figure 17:**
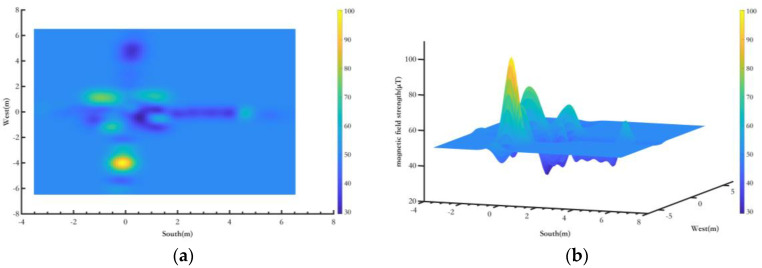
Magnetic landmark point magnetic fingerprint database. (**a**) Magnetic landmark top view; (**b**) Magnetic landmark three−imensional view.

Third, the magnetic landmark fingerprint constructed for indoor Route 2 is shown in Figure 17. Figure 17 shows the magnetic landmark fingerprint of point A on Route 2.

Figure 17a shows the planar diagram of a magnetic landmark fingerprint at one point, and Figure 17b shows the three-dimensional diagram of the magnetic landmark fingerprint. Due to the complex spatial environment of the test area, the magnetic field environment is also quite intricate, which may result in significant magnetic field variations. However, since the objects in the space remain stationary over long periods, the environmental magnetic field remains stable over time and can be used for magnetic field matching. The magnetic landmark fingerprint provides a solid foundation for the pedestrian pose optimization method based on magnetic landmark matching proposed in Section 3. Due to space limitations, only the magnetic fingerprint map for one magnetic landmark is presented in this section.

**Figure 18:**
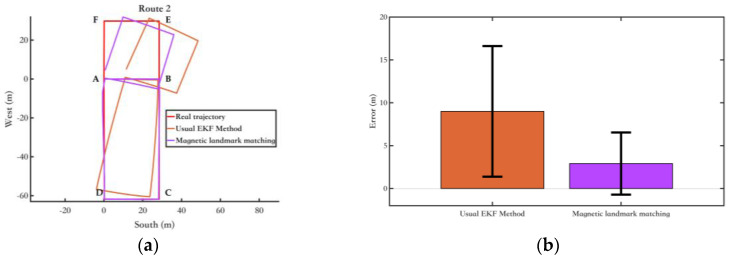
Experimental results of the fusion algorithm proposed in this section. (**a**) Route 1 calculated by the 2 methods; (**b**) The position error bars of different methods. The range indicated by the black line represents the RMS value of the corresponding error.

Finally, the walking experiment was conducted on Route 2, which has a total length of 297.08 m. The magnetic landmark matching method proposed in Section 3 was compared with the EKF method using only single inertial sensor. The experimental results are as follows:

As shown in Figure 18a, points A, B, C, D, E, and F in the diagram are magnetic landmark points. In the ABCD segment, the magnetic landmark matching is good, and the pedestrian walking trajectory almost overlaps with the true trajectory. However, in the BE, EF, and FA segments, the pedestrian position shows a shift, which is due to matching errors at the magnetic landmark points B, E, and F. This led to errors in the pose calibration of the pedestrian for these three segments, ultimately causing the trajectory distortion. However, as shown in Figure 18b, the method based on ordinary EKF + magnetic landmark matching significantly reduces the pedestrian trajectory estimation system error, improving overall positioning accuracy.

### 4.3. Fusion Method Experimental Validation

The methods proposed in this paper are categorized and summarized, and experimental verification is conducted using Experiment route 1 and Experiment route 2 in this section.

- Method 1: Use ordinary EKF combined with the ZUPT algorithm;
- Method 2: Use the EKF method combining inertial navigation and the magnetic field, as described in Section 2.2;
- Method 3: The yaw angle variable constraint was added to Method 2, as described in Section 2.3;
- Method 4: The magnetic landmark matching-based pose optimization method was added to Method 1;
- Method 5: The magnetic landmark matching-based pose optimization method was added to Method 3, which is the overall pedestrian localization method proposed in this paper.

First, the constraints on the heading angle for the methods proposed in this paper are verified. Experiment route 1 is applicable for Method 1 to Method 3, while Experiment route 2, which includes magnetic landmarks, is applicable for Method 1 to Method 5. This section provides error probability distribution and error bars, as well as the average error, root-mean-square error, maximum error, and drift rate (average error divided by total duration) for each method.

**Figure 19:**
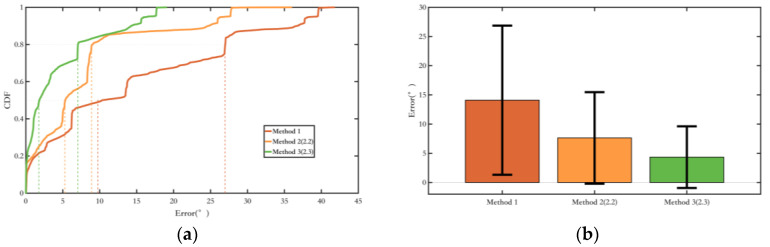
Comparison of yaw angle error in route 1. (**a**) Error probability distribution of each method; (**b**) Error bars of each method. The range indicated by the black line represents the RMS value of the corresponding error.

Figure 19 shows the comparison of yaw angle errors in route 1, and Table 4 presents the quantitative comparison of yaw angle errors. Methods 2 and 3, by introducing new magnetic field measurements, show better suppression of heading angle errors compared to Method 1. Method 3, which adds a constraint on the heading angle change during pedestrian turns, achieves better overall heading angle error suppression than Method 2, making more efficient use of the environmental magnetic field information.

**Figure 20:**
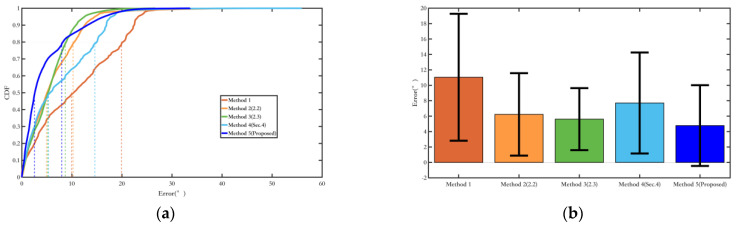
Comparison of yaw angle error in route 2. (**a**) Error probability distribution of each method; (**b**) Error bars of each method. The range indicated by the black line represents the RMS value of the corresponding error.

Figure 20 shows the comparison of yaw angle errors in route 2, and Table 5 presents the quantitative comparison of yaw angle errors. Although the indoor magnetic field environment is more complex, the magnetic field fusion methods, Methods 2 and 3, still yield good results. Compared to Experiment route 1, the suppression of heading angle errors is more apparent in Experiment route 2. This is because the walking distance is longer, and in Method 1, which uses only the ordinary EKF, the cumulative yaw angle error increases. However, Methods 2 and 3, by incorporating magnetic field measurements, are able to suppress the cumulative error effectively. Method 4, compared to Method 1, shows good results in yaw angle error suppression after adding magnetic landmark matching. Still, due to errors in magnetic landmark matching, its overall yaw angle accuracy is not as good as those of Methods 2 and 3. Method 5, based on Method 3 with added magnetic landmark matching, benefits from better yaw angle error suppression in Method 3 compared to Method 1, which increases the probability of successful matching, resulting in the best yaw angle error suppression.

Finally, the constraints on displacement using the proposed method in this paper are validated. The corresponding trajectory and comparison of positioning error are provided, along with the detailed average error, root-mean-square error, maximum error, and relative average error. The calculation method for relative average error is the average error divided by the total walking distance. This is used to more accurately describe the overall performance of the method, rather than just using average error to describe performance while ignoring the error accumulation brought by the total walking distance.

**Figure 21:**
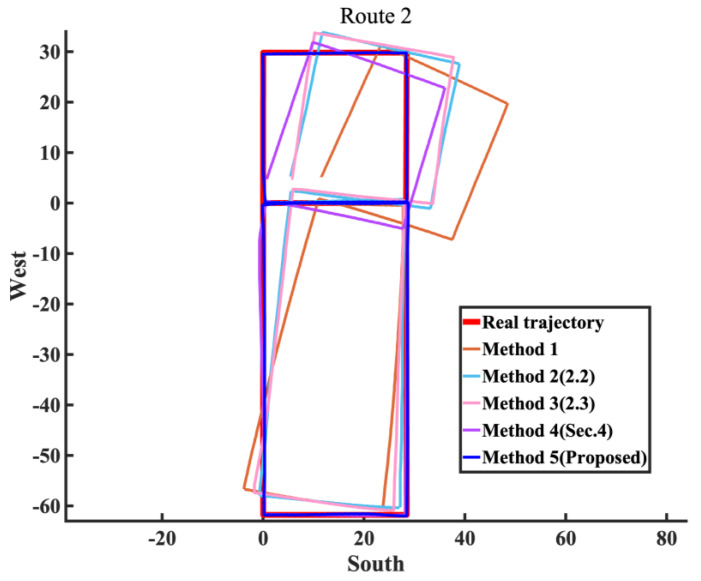
Route 2 calculated by the 5 methods.

Similarly to the yaw angle error, due to the longer walking distance in Experiment Route 2, the advantages of Methods 2 and 3, which combine inertial and magnetic field fusion for heading angle estimation, are more evident, thereby significantly improving their overall localization performance compared to Method 1. Although Method 4 has three magnetic landmarks that were not matched, its overall localization error is still controlled within a small range, with a relative average error of 0.98%. Since Method 5 is Method 3 with added magnetic landmark matching, and Method 4 is Method 1 with added magnetic landmark matching, it is clear that the fusion algorithm in Section 2 greatly enhances the overall positioning accuracy while also increasing the probability of successful magnetic landmark matching. Comparing Method 5 with Method 3, as shown in Figure 21, it is evident that the trajectory of Method 5 almost coincides with the true trajectory. This reflects the superiority of the matching algorithm in Section 3, resulting in a one-order-of-magnitude improvement in the relative average error for Method 5, bringing it to a level of 0.12% (Table 6).

**Figure 22:**
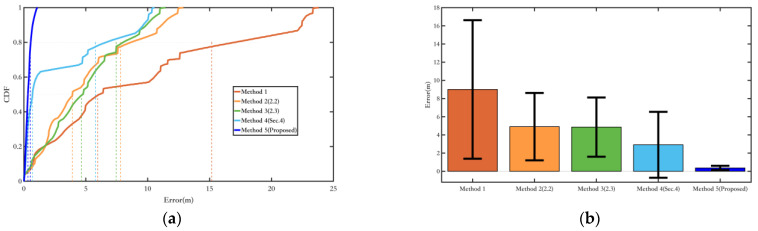
Comparison of positioning error. (**a**) Comparison of positioning error probability distribution of each method; (**b**) Comparison of positioning error bars of each method. The range indicated by the black line represents the RMS value of the corresponding error.

The experiments above validate the effectiveness of the proposed method in both quasi-indoor and indoor environments. The method significantly reduces yaw angle and position errors, eliminates cumulative historical errors, and produces more accurate pedestrian trajectories (Figure 22).

**Figure 23:**
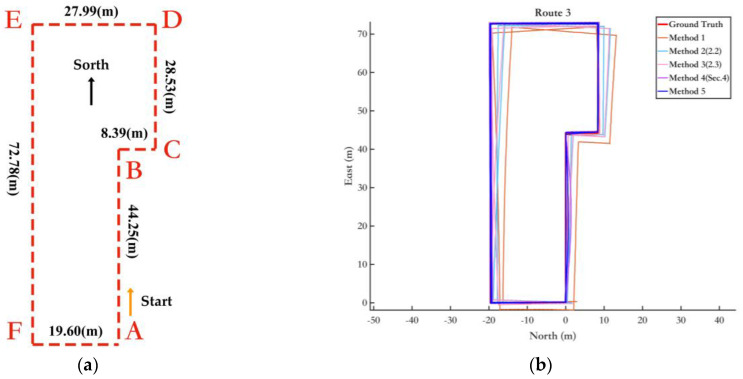
Ground truth of Experiment route 3. (**a**) Comparison of positioning error probability distribution of each method; (**b**) Route 3 calculated by the 5 methods.

To further verify the effectiveness of the proposed fusion method in different indoor environments, we conducted validation experiments in a new indoor setting. Experimental route 3, in the new indoor environment, is shown in Figure 23a, and the pedestrian trajectories obtained by different methods are shown in Figure 23b.

In the indoor environment of Route 2, classrooms occupy the majority of the space. In contrast to Route 2, Route 3 was selected in an indoor setting with more laboratories. Due to the presence of various metals and electronic devices in the laboratories, the experiment on Route 3 is more challenging. According to Figure 23a, the pedestrian route in this experiment follows the path A-B-C-D-E-F, forming two complete closed loops with a total distance of 403.8 m. As shown in the trajectory plot in Figure 23b, the blue portion representing the proposed fusion method still demonstrates excellent performance, exhibiting a very high degree of alignment with the ground truth trajectory.

**Figure 24:**
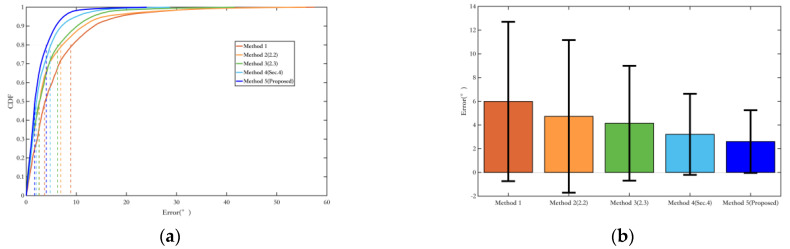
Comparison of yaw angle error in route 3. (**a**) Error probability distribution of each method; (**b**) Error bars of each method. The range indicated by the black line represents the RMS value of the corresponding error.

Figure 24 shows the comparison of yaw angle errors in route 3, and Table 7 presents the quantitative comparison of yaw angle errors.

It is evident that the proposed Methods 2, 3, and 5 in this paper show progressively improved optimization results in terms of both the average error and the RMS error, which represents data fluctuations. Although the environment of route 3 has more magnetic interference, the incorporation of magnetic field observations in Methods 2 and 3 overall enhances the positioning performance. Moreover, Method 5, which fuses magnetic heading with magnetic field matching, makes more effective use of the magnetic field information, resulting in better yaw angle optimization. Compared with the EKF filtering method using only a single inertial sensor, the error is reduced by over 50%.

**Figure 25:**
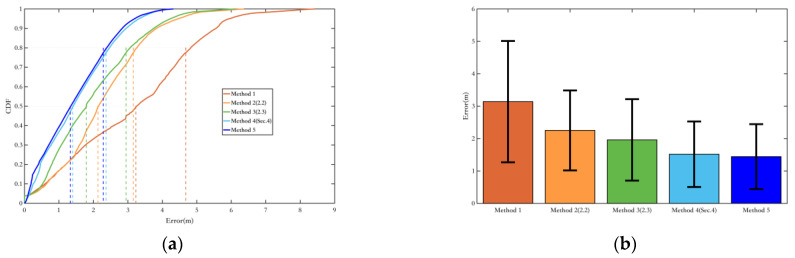
Comparison of positioning error in route 3. (**a**) Comparison of positioning error probability distribution of each method; (**b**) Comparison of positioning error bars of each method. The range indicated by the black line represents the RMS value of the corresponding error.

Similarly to the yaw optimization results, the proposed Method 5 in this paper can fully utilize both quasi-static and non-quasi-static magnetic field information to directly or indirectly optimize the position. The pose optimization method based on magnetic landmark matching can greatly reduce position errors by eliminating cumulative historical errors (Figure 25). Compared with the EKF filtering method using only a single inertial sensor, the position error is reduced by more than 50% (Table 8).

## 5. Conclusions

The main research content of this paper focuses on a high-precision pedestrian localization method based on the fusion of inertial and magnetic field data. In complex indoor environments, satellite navigation technology performs poorly, and infrastructure-based indoor navigation technologies are expensive, which has led to the development of autonomous pedestrian navigation technology based on MEMS sensors. Pedestrian trajectory estimation methods based on pure inertial sensors lack external observations that provide absolute information, resulting in cumulative errors and distorted estimated trajectories. To address this issue, this paper uses magnetic field information to provide reliable external observations, thereby suppressing the cumulative errors in the overall pedestrian localization system and achieving higher positioning accuracy.

First, this paper proposes a method for constraining pedestrian yaw angles by fusing magnetic field information with inertial navigation. The EKF-based yaw angle fusion algorithm is improved by integrating quasi-static determination into the filter and estimating the reliability of the magnetic field using the covariance matrix, thus enhancing the accuracy of the fusion algorithm. A yaw angle variable constraint based on the magnetic field yaw angles is introduced, and a pedestrian yaw angle variable constraint method is proposed by utilizing the pedestrian’s state during turns. This method expands the use range of the fusion algorithm and further suppresses heading angle errors. Secondly, a trajectory optimization method is proposed based on magnetic landmark matching for error suppression and pose constraints. A magnetic field matching method is introduced, taking advantage of the sharp changes in non-quasi-static magnetic fields, maximizing the utilization of both quasi-static and non-quasi-static information from the environmental magnetic field. Collecting environmental magnetic field information and constructing a magnetic landmark field map, a trajectory optimization method is proposed using magnetic field matching based on magnetic landmarks and pose graph optimization. This method clears accumulated errors and optimizes poses. Only a small amount of magnetic field information is collected to form magnetic landmarks. Landmark identification is performed using the ICCP magnetic field matching method, and pose graph optimization maximizes the use of magnetic landmarks to optimize the overall trajectory, further suppressing displacement errors. Finally, the fusion yaw angle constraint method and magnetic landmark matching trajectory optimization method fully utilize quasi-static and non-quasi-static magnetic field information. The yaw angle constraint method provides a higher success rate for magnetic landmark matching trajectory optimization. And the magnetic landmark matching trajectory optimization method improves the overall trajectory optimization, achieving excellent results.

This paper conducts experimental comparison and verification of the proposed methods from two aspects: yaw angle and trajectory estimation accuracy, in both quasi-indoor and indoor environments. The results show that constraining pedestrian yaw angles by fusing magnetic field information with inertial navigation can significantly reduce yaw angle errors. In quasi-indoor environments with relatively small magnetic field interference, the average error and drift rate were reduced by nearly 70%. The average error and drift rate were reduced by nearly 50% in purely indoor environments with higher magnetic field interference. Combined with the magnetic landmark matching-based cumulative error removal and pose optimization method, further suppression of heading errors and a significant reduction in cumulative position errors were achieved. In purely indoor environments with greater magnetic field interference, the average yaw angle error and drift rate decreased by nearly 60%, and the overall algorithm’s relative positioning error was reduced by 96%, resulting in excellent localization performance.

For discussion and comparison, reference [36] proposed a smartphone-based indoor positioning algorithm that fuses magnetic field and inertial data. Ref. [36] generated a gridded magnetic fingerprint map using smartphone measurements and performed magnetic field matching with an extended two-dimensional matching method based on DTW, fusing the smartphone’s inertial data for indoor positioning. Over a walking distance of 78 m, they achieved a relative average error of 0.9%, representing an improvement of up to approximately 97% over traditional PDR methods. The advantage of the method in [36] is its convenience in performing indoor positioning using a smartphone. In contrast, the method proposed in this paper achieves a relative average error of up to 0.4% in long-distance walking over 200 m, with a maximum improvement of about 97% compared to traditional methods. Compared with [36], the optimization effect of our method is in the same order of magnitude, with slightly better results than [36]. Our advantage lies in the ability to achieve high-precision pedestrian indoor positioning during long-term and long-distance walking.

At the same time, there are still some shortcomings and areas for further optimization in this paper:

(1) The application scenario in this paper is limited to normal pedestrian walking. In future research, the movement characteristics of pedestrians in different scenarios will be analyzed to expand the applicability of the proposed method.
(2) This paper focuses only on a two-dimensional plane, and the pose graph method is also analyzed within the two-dimensional plane. In future research, the dimensions of the pose graph will be further expanded to study pedestrian navigation and localization in buildings, thereby broadening the application scenarios.

In summary, this paper proposes a pedestrian indoor localization method that maximizes the use of environmental magnetic field information by fusing inertial navigation and magnetic field data. The method utilizes quasi-static magnetic field information for heading constraint and non-quasi-static information for magnetic landmark matching to eliminate historical errors and optimize pedestrian poses. The position error was reduced by one order of magnitude in the overall localization experiments, achieving great research results.

## Figures and Tables

**Table 1:** Advantages and limitations of non-autonomous positioning methods.

Positioning Method	Advantage	Disadvantage
Global Positioning System	High accuracy in open environment	Weak signal in urban or indoor environments
Bluetooth	High popularity, lightweight, and low power consumption	Requires additional indoor infrastructure
WIFI	Widely used, low hardware cost	Requires additional indoor infrastructure, Easily affected by complex RSS environments
UWB	High time resolution, high accuracy, low power consumption	Existence of multipath effects, Existence of non-line-of-sight errors, The overall cost is relatively high
Infrared technology	Higher positioning accuracy	Easily affected by complex environmental interference
Ultrasonic technology	Higher positioning accuracy	High cost and easily affected by complex environmental interference

**Table 2:** Key event segmentation of the pedestrian gait cycle.

Key Events	HS	SS	MS	HO	TO	FO	VP	HS
Gait Phases	Initial Contact	Flatfoot (30%)		Pedal and Stretch (20%)		Swing (40%)		
	Stance (60%)					Swing (40%)		
	Double Support	Single Support on Right Foot			Double Support	Single Support on Left Foot		
	Right Step (50%)				Left Step (50%)			
Barycenter	Lowest	Moving up	Highest	Moving down	Lowest	Moving up	Highest	Moving down

**Table 3:** HWT905-TTL sensor specifications.

Index	Three-Axis Accelerometer	Three-Axis Gyroscope	Three-Axis Magnetometer
Range	±1.5 g	±2000°/s	±800 T
Zero Offset	±20 mg	±0.5~1°/s	
Resolution	0.0005 (g/LSB)	0.061 (°/s)/(LSB)	13 (nT/LSB)
sampling frequency	100 Hz	100 Hz	100 Hz

**Table 4:** Quantitative comparison of yaw angle errors in Route 1.

	Method 1	Method 2	Method 3
MEAN (°)	14.094	7.631	4.339
RMS (°)	12.771	7.839	5.279
MAX (°)	41.787	35.981	19.043
50% (°)	12.139	5.456	1.926
80% (°)	27.018	9.301	7.139
DRIFT (°/s)	0.127	0.069	0.039

**Table 5:** Quantitative comparison of yaw angle errors in route 2.

	Method 1	Method 2	Method 3	Method 4	Method 5
MEAN (°)	11.043	6.228	5.612	7.706	4.778
RMS (°)	8.229	5.344	4.020	6.546	5.235
MAX (°)	55.593	50.532	26.662	55.843	33.585
50% (°)	10.690	5.192	5.373	6.000	2.657
80% (°)	20.591	10.721	8.994	15.115	8.426
DRIFT (°/s)	0.024	0.014	0.012	0.017	0.011

**Table 6:** Quantitative comparison of positioning errors in route 2.

	Method 1	Method 2	Method 3	Method 4	Method 5
Mean (m)	8.999	4.908	4.858	2.912	0.364
RMS (m)	7.618	3.706	3.256	3.624	0.238
MAX (m)	23.819	12.883	11.446	10.553	1.100
50% (m)	6.255	3.927	4.812	0.692	0.322
80% (m)	18.969	9.638	8.711	7.695	0.580
Relative Average (%)	3.02	1.65	1.63	0.98	0.12

**Table 7:** Quantitative comparison of yaw angle errors in route 3.

	Method 1	Method 2	Method 3	Method 4	Method 5
MEAN (°)	5.983	4.729	4.148	3.213	2.603
RMS (°)	6.719	6.435	4.841	3.418	2.645
MAX (°)	57.494	55.734	41.502	28.614	23.987
50% (°)	3.911	2.751	2.771	2.115	1.774
80% (°)	9.579	7.595	6.837	5.073	4.313
DRIFT (°/s)	0.009	0.007	0.007	0.005	0.004

**Table 8:** Quantitative comparison of positioning errors in route 3.

	Method 1	Method 2	Method 3	Method 4	Method 5
Mean (m)	3.140	2.252	1.959	1.517	1.442
RMS (m)	1.872	1.234	1.257	1.011	1.003
MAX (m)	8.419	6.371	6.102	4.251	4.320
50% (m)	3.291	2.164	1.800	1.427	1.360
80% (m)	4.975	3.368	3.219	2.564	2.484
Relative Average (%)	0.008	0.006	0.006	0.004	0.004

## Data Availability

The original contributions presented in this study are included in the article. Further inquiries can be directed to the corresponding author.

## Abbreviations

The following abbreviations are used in this manuscript:

IMU	Inertial Measurement Unit
EKF	Extended Kalman Filter
UWB	Ultra-Wideband
RFID	Radio Frequency Identification
MEMS-IMU	Micro-Electro-Mechanical System Inertial Measurement Unit
SINS	Strapdown Inertial Navigation Systems
PDR	Pedestrian Dead Reckoning
ZUPT	Zero-Velocity Update
QZVI	Quasi-Zero-Velocity Interval
OGC	One-Gait-Cycle
ZVI	Zero-Velocity Interval
QSMF	Quasi-Static Magnetic Field
IPF	Integrating Particle Filter
PF	Particle Filter
NLOS	Non-Line-Of-Sight
DTW	Dynamic Time Warping
DCM	Direction Cosine Matrix
HS	Heel Strike
SS	Sole Strike
MS	Mid Support
HO	Heel Off
TO	Toe Off
FO	Foot Off
VP	Vertical Position
MM	Magnetic Field Matching
ICCP	Iterative Closest Contour Point
ICP	Iterative Closest Point
PGO	Pose Graph Optimization
SLAM	Simultaneous Localization and Mapping
